# Proteomic Analysis of Fetal Ovary Reveals That Ovarian Developmental Potential Is Greater in Meishan Pigs than in Yorkshire Pigs

**DOI:** 10.1371/journal.pone.0135514

**Published:** 2015-08-25

**Authors:** Mengmeng Xu, Long Che, Dingyue Wang, Zhenguo Yang, Pan Zhang, Yan Lin, Zhengfeng Fang, Lianqiang Che, Jian Li, Daiwen Chen, De Wu, Shengyu Xu

**Affiliations:** 1 Key Laboratory for Animal Disease Resistance Nutrition of the Ministry of Education of China, Animal Nutrition Institute, Sichuan Agricultural University, Ya’an, 625014, China; 2 Tequ Group of Sichuan Province, Chengdu, 610207, China; Zhejiang University, CHINA

## Abstract

Time-dependent expression of functional proteins in fetal ovaries is important to understand the developmental process of the ovary. This study was carried out to enhance our understanding of the developmental process of porcine fetal ovaries and to better address the differences in fetal ovary development of local and foreign pigs. The objective of the present study is to test the expression of key proteins that regulate the growth and development of fetal ovaries in Meishan and Yorkshire porcine breeds by using proteomics technology. Six Meishan and 6 Yorkshire pregnant gilts were used in this experiment. Fetal ovaries were obtained from Yorkshire and Meishan gilts on days 55 and 90 of the gestation period. Using 2D-DIGE (two dimensional-difference in gel electrophoresis) analysis, the results showed that there are about 1551 and 1400 proteins in gilt fetal ovaries on days 55 and 90, respectively of the gestation. Using MALDI TOF-TOF MS analysis, 27 differentially expressed proteins were identified in the fetal ovaries of the 2 breeds on day 55 of gestation, and a total of 18 proteins were identified on day 90 of gestation. These differentially expressed proteins were involved in the regulation of biological processes (cell death, stress response, cytoskeletal proteins) and molecular functions (enzyme regulator activity). We also found that alpha-1-antitrypsin, actin, vimentin, and PP2A proteins promote the formation of primordial follicles in the ovaries of Yorkshire pigs on day 55 of gestation while low expression heat shock proteins and high expression alpha-fetoproteins (AFP) may promote Meishan fetal ovarian follicular development on day 90 of gestation. These findings provide a deeper understanding of how reduced expression of heat shock proteins and increased expression of AFP can significantly reduce the risk of reproductive disease in obese Meishan sows. Our study also shows how these proteins can increase the ovulation rate and may be responsible for the low reproductive efficiency reported in other obese breeds. The ovarian developmental potential was found to be greater in Meishan pigs than in Yorkshire pigs.

## Introduction

Reproductive productivity in the female pig is determined by follicle assembly, development, oocyte numbers, and quality. However, the activation amount of primordial follicles to growing follicles and the incidence of atresia in oocytes are also important to determine the lifespan of ovaries [1]. In the embryonic phase, the pig ovary contains a finite number of oocytes [2]. Accordingly, proper regulation of the initial oocyte number is critical for the ovary to maintain long-term fertility [3]. It is now well established that the primordial follicle pool can be a major determinant of female mammalian reproductive life, although some studies have implied that germ line stem cells exist in the adult ovary [4]. Primordial follicle formation and activation is irreversible, where the number of primordial follicles allotted to female mammals at birth is a limited number [5]. Moreover, any abnormalities in the development of ovary cells can further reduce follicle numbers, even leading to infertility [6]. However, the underlying mechanism for embryo ovary development still needs to be investigated.

A limited number of proteins control diverse biological processes, including development, differentiation and apoptosis in specific organisms at specific times [7]. Recently, there has been some progress in understanding the molecular mechanisms of ovary development in European breeds and local pig breeds of China. Studies using microarray profiling have identified several genes that contribute to the differential development of the ovaries of two divergent breeds of pigs [8]. Although a few studies have focussed on revealing novel factors contributing to Meishan and Yorkshire pig fetal ovary development, the mechanism of protein regulation of ovary development remains largely unexplored. Identifying the proteins in Meishan and Yorkshire pigs will provide molecular insight into the differences in fetal ovary development. Unfortunately, there is little information to meet this significant challenge.

In European porcine ovaries, germ cells begin to enter meiosis on day 47 of gestation and follicles begin to form at approximately day 56 of gestation [9]. However, the primordial follicle that was first detected in local pig breeds in China was on day 70 of gestation [10]. We were particularly intrigued that the transition to the primary stage occurs in the late gestational period of day 90 in two divergent breeds. Therefore, the objective of the present study was to test the hypothesis that the key proteins that regulate growth and development of fetal ovaries are different in Meishan and Yorkshire pigs.

## Materials and Methods

### Gilts and tissue sample collection

All experimental procedures conducted in this study were approved by the Guide for the Care and Use of Laboratory Animals prepared by the Animal Care and Use Committee of Sichuan Agricultural University. Six Meishan and six Yorkshire healthy gilts were used in this experiment. Meishan or Yorkshire gilts of the same genetic background (derived by breeding the same respective parental stocks and same parity) were kept in the same farm conditions with temperature, humidity and feeding-time. Meishan gilts with similar weights (72.84 ± 0.66 kg) were checked for oestrous behavior. The gilts were artificially inseminated twice with sperm from an identical Meishan boar. Similarly, six Yorkshire gilts at about 135.54 ± 0.66 kg were mated at the 3^rd^ estrus with identical Yorkshire boars. After artificial insemination, the pregnant gilts were housed individually and fed 2 kg/d (0–30 d) or 2.4 kg/d (31–90 d). The energy intake was 3.4 Mcal DE/kg for Yorkshire gilts and 3.0 Mcal DE/kg for Meishan gilts. The diet formulated to meet nutrient requirements was recommended by the National Research Council (1998) for gestational gilts (crude protein—13.9%, Lys—0.69%, calcium—0.96% and phosphorus—0.79%). All gilts had *ad libitum* access to water. Three Meishan and three Yorkshire pregnant gilts were randomly selected on day 55 to be anaesthetized with an intravenous injection of Zoletil 50 (0.1 mg/kg body weight; Zoletil 50 Vet, Virbac, Carros cedex, France), and were slaughtered. The remaining pregnant gilts were similarity slaughtered on day 90 of gestation. The female fetal ovaries of the gilts were immediately excised. Dissected samples of each fetal ovary were rapidly placed in liquid nitrogen and stored at -80°C until use.

### Protein sample preparation

The ovary samples were obtained from 4 groups: Meishan gilts on day 55 of gestation, Meishan gilts on day 90 of gestation, Yorkshire gilts on day 55 and Yorkshire gilts on day 90 of gestation, respectively. Each group consisted of 3 replicates that were obtained from 3 pregnant gilts. Each replicate sample was a pooled sample from 3 randomly selected fetal ovary tissues samples of 1 pregnant gilt. Frozen ovaries were crushed with a mortar and pestle in liquid nitrogen. These powders were then mechanically homogenised and dissolved in lysis buffer containing 2 M thiourea (Guangzhou Chemical Reagent Factory, Guangzhou, China), 7 M urea (Guangzhou Chemical Reagent Factory, Guangzhou, China), phenylmethanesulfonyl fluoride (PMSF, Sangon Biotech (Shanghai) Co., Ltd., Shanghai, China), and 4% w/v CHAPS (Sangon Biotech (Shanghai) Co., Ltd., Shanghai, China) on ice. These homogenates were sonicated intermittently and then separated by centrifugation at 13,000 × g for 20 min at 4°C. The supernatant was obtained and acetone (Sigma-Aldrich Co. LLC., MO, USA) was added to the supernatant. The mixture was centrifugated and separated at 13 000 × g for 20 min at 4°C. The precipitate was harvested and stored at -80°C. The protein extracted from each group was prepared for two dimensional-difference in gel electrophoresis (2D-DIGE) analysis. The concentration of the protein was determined using a 2D Quant kit (Amresco, LLC, OH, USA)

### Protein sample labelling and two dimensional-difference in gel electrophoresis

Two dimensional-difference in gel electrophoresis of protein samples was performed in duplicate. The internal standard pool of each gel was generated by combining equal amounts of proteins from all the synchronized samples of either Meishan or Yorkshire pigs (day 55 or day 90), and was labelled with Cy2. Protein extracts from the fetal ovaries of Meishan day 55 and Yorkshire day 55 treatments or Meishan day 90 and Yorkshire day 90 treatments were labelled with Cy3 and Cy5, respectively. The different dyes were mixed with 1% dithiothreitor (Guangzhou Chemical Reagent Factory, Guangzhou, China), 1% IPG (immobilised pH gradient) buffer (GE Healthcare Life Sciences, NJ, USA), rehydration buffer (bromopheol blue (Guangzhou Chemical Reagent Factory, Guangzhou, China) in 7 M urea, 2 M thiourea, 4% PMSF) 460 μL. The mixture was loaded onto 24 cm strips, with a 4–7 linear pH IPG dry strip. For the 2-dimensional (2D) fluorescence difference in gel electrophoresis, an IPG strip was loaded with a protein mixture for each gel after 12 hours of rehydration. The protein mixture was subjected to isoelectric focusing (IEF) and proteins were subsequently separated by sodium dodecyl sulphate-polyacrylamide gel electrophoresis (SDS-PAGE). Prior to SDS-PAGE, the IPG strips were equilibrated in an 8 mL equilibration solution containing 30% glycerol, 0.002% bromopheol blue, 6 M urea, 4% SDS, and subsequently mixed with 100 mM dithiothreitor for 15 min. The strips were cut and 250 mM iodoacetamide (Guangzhou Chemical Reagent Factory, Guangzhou, China) was added to the same solution for 15 min. The specific setting method is as previously described [11], with some modifications. Briefly, first dimension electrophoresis was performed with the following program: 300 V for 30 min, 700 V for 30 min, 1500 V for 1.5 h, 9000 V for 3 h, and, finally, 9000 V for 4 h. The electrophoresis was set at 52 KVh. Second dimension electrophoresis was used to separated the proteins with the following settings: 2 W/gel for 45 min and then at 17 W/gel for 4.5 h until bromophenol blue reached the bottom of the gel.

### Gel image acquisition and analysis

The Typhoon 9400 imager (GE Healthcare Life Sciences, NJ, USA) was used to scan for proteins at wavelengths of 488/550 nm (cy2), 532/550 nm (cy3), and 633/550 nm (cy5), respectively. Image Master 2D platinum 7.0 software (GE Healthcare Life Sciences, NJ, USA) was used to analyze the 2D- DIGE gel images. The calculated protein abundance changes for spot picking were detected using Cy3/Cy2 and Cy5/Cy2 differential in-gel analysis ratios. The significant differences in protein spot changes (P < 0.05, ratio_Yorkshire/Meishan_ > 1.6 or ratio_Meishan/Yorkshire_ > 1.6 form paired t test statistical analysis of the data) were selected for excision of protein spots for further analysis.

### Spot picking and enzymatic digestion

Selected spots were automatically cut form gels, treated and, washed twice with Milli-Q water for 30 min in 50% methyl alcohol (Guangzhou Chemical Reagent Factory, Guangzhou, China) until destained. The spots were dissolved in 100 mM NH_4_HCO_3_ (Sigma-Aldrich Co. LLC, MO, USA) and extracted with 50% acetonitrile (ACN; Sigma-Aldrich Co. LLC., MO, USA). The spots were then digested with 1 μg/μL robust trypsin (Promega Corporation, WI, USA) for 30 min. Coverage solution (10% ACN, Milli-Q water, 50 mM NH_4_HCO_3_) was then added, following incubation at 16 h. Following digestion, the generated peptides were extracted with an appropriate solvent that was 90% ACN and 2.5% trifluoroacetic acid (TFA; Promega Corporation, WI, USA) for 30 min. Finally, the peptides were vacuum dried.

### Matrix-assisted laser desorption ionization time-of-flight mass spectrometry (MALDI-TOF-TOF MS) analysis

After vacuum drying, the peptide material was dissolved in 30% ACN, 0.1% trifluoroacetic acid (TFA), and Milli-Q water in a volume of 1.5 μL. Then 0.8 μL of the mixture with 0.5 μL of 5 mg/mL α-cyano-4-hydroxycinnamic acid (50% ACN containing 0.1% TFA) was loaded onto a metal sample plate and then dried at room temperature. Finally, the MALDI-TOF-TOF (Bruker Daltonik GmbH, Bremen, Germany) was used to perform MS analysis. The UA laser conditions were setat 355 nm wavelength, 200 Hz repetition rate, and 30 kV accelerated voltage. A flexAnalysis (Bruker Daltonik GmbH, Bremen, Germany) was used to filter the baseline peak and to identify the signal peak. The following search, NCBI database BioTools (Bruker Daltonik GmbH, Bremen, Germany) was used to analyze a unique identification code, the values of sequence coverage, cleavage of trypsin, oxidation of variable modifications, the relative molecular mass, and the PI value. All the queries were performed with the following settings: 800–4000 Da molecular range of peptides; apparent pI and apparent Mr error range unlimited; maximum missed cleavage 1; 50 ppm peptide tolerance; fragment mass tolerance within 0.6 Da. In this study, the selected protein had a feature that is a significant match (P < 0.05) and a score higher than 89. We also identified and analyzed proteins within the KEGG (Kyoto Encyclopedia of Genes and Genomes) metabolic pathway maps (http://www.kegg.jp/).

### Statistical analysis

Data are expressed as mean and standard deviation. Data were statistically analyzed with t test, using software provided by ImageMaster 2D platinum 7.0 (GE Healthcare Life Sciences, NJ, USA). Proteins were identified using MASCOT and (http://www.matrixscience.com) and BioTools (Bruker Daltonik GmbH, Bremen, Germany). P < 0.05 was considered significant.

## Results

### 2D-DIGE analysis and identification of differentially expressed proteins

**Fig 1:**
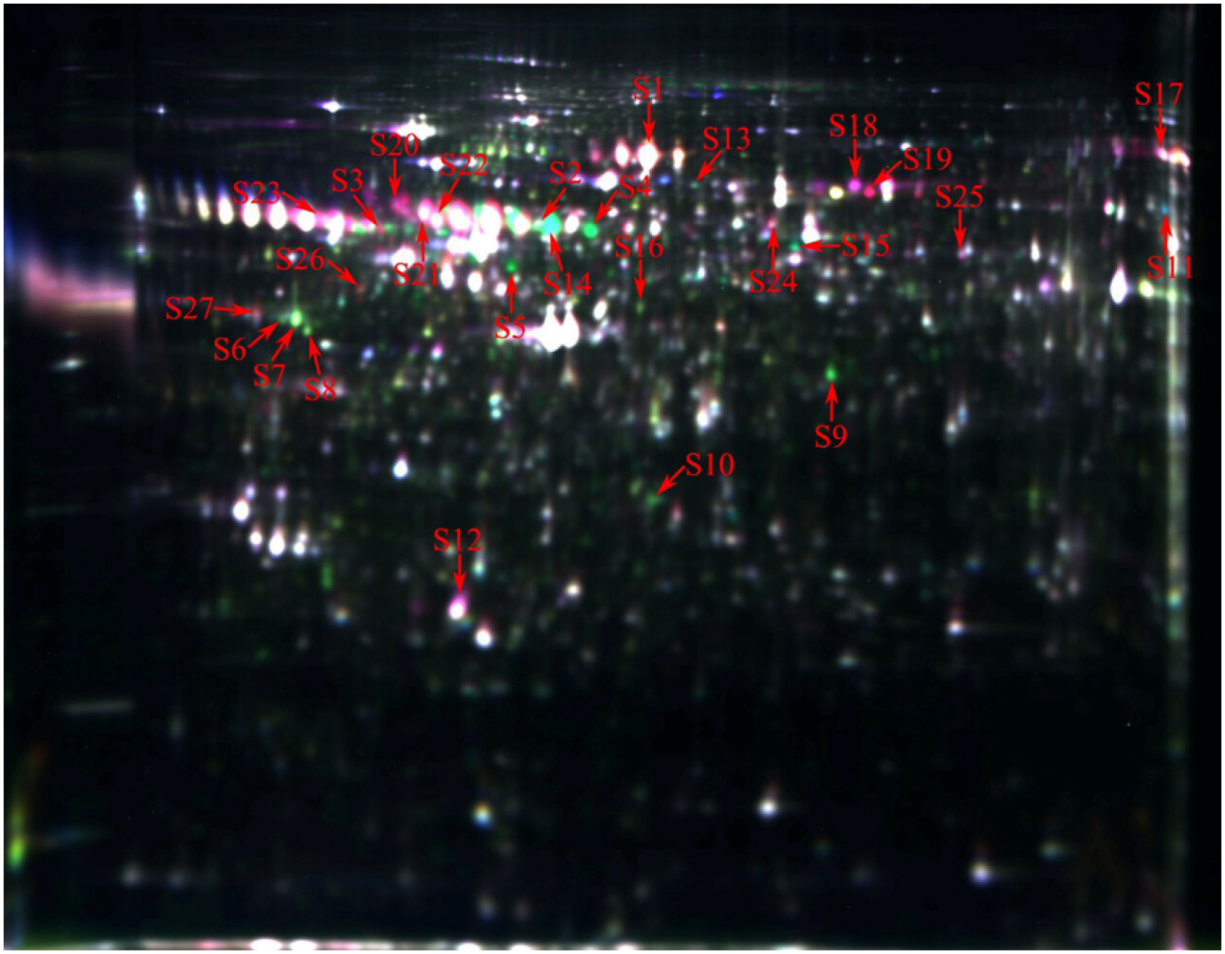
Representative DIGE gel image of differentially expressed proteins in Yorkshire and Meishan fetal ovaries at gestation day 55. The proteins extracted from the Yorkshire and Meishan fetal ovaries on day 55 of gestation samples were labelled with Cy3 and Cy5, respectively. An internal standard protein sample (a mixture of Yorkshire and Meishan fetal ovaries on day 55 of gestation samples) was labeled with the Cy2 dye. The S2-S11 and S12-S16 spots represent up-regulated proteins, while the others spots represent down-regulated proteins in the in Yorkshire fetal ovaries samples compared with the Meishan fetal ovaries samples. The number in the figure corresponds to the number shown in Table 1.

About 1551 protein spots were detected and 62 individual protein spots were significantly changed (ratio_Yorkshire/Meishan_ > 1.5 or ratio_Meishan/Yorkshire_ > 1.5, P < 0.05) on day 55 of gestation in Yorkshire and Meishan fetal ovaries. Twenty seven differentially expressed proteins (ratio_Yorkshire/Meishan_ > 1.6 or ratio_Meishan/Yorkshire_ > 1.6, P < 0.05) were selected for MALDI TOF-TOF MS analysis (Fig 1). The proteomic analysis shows that Yorkshire fetal ovaries had 14 upregulated proteins and 13 downregulated proteins compared with Meishan fetal ovaries at day 55 of gestation (Table 1). Yorkshire fetal ovaries express proteins that regulate primordial follicle assembly, such as actin, alpha-1-antitrypsin, vimentin, while Meishan fetal ovaries have more proteins play role on uncleaved oocyte stage, such as serine/threonine-protein phosphatase 2A 65 kDa regulatory subunit A beta isoform and, serine/threonine-protein phosphatase 2A 65 kDa regulatory subunit A alpha isoform.

**Table 1:** Differential proteins between Yorkshire and Meishan fetal ovaries on day 55 of gestation identified by MALDITOF/TOF.

No.	Protein-IDs	Moscot score	Mr	PI	Fold change	Coverage	Protein name
S1	FETA	390	70405	5.47	-2.0225	30%	Alpha-fetoprotein
S2	F1SMZ7	162	61266	5.61	+1.80537	20%	Uncharacterized protein
S3	F1RIP6	188	53170	5.02	+2.02844	30%	Uncharacterized protein
S4	I3LQS0	89	49512	5.38	+9.00958	14%	Uncharacterized protein
S5	F1SME6	331	56996	6.00	+9.00958	28%	Uncharacterized protein
S6	F1SGP8	169	38756	4.74	+1.62898	24%	Uncharacterized protein
S7	I3LMU6	194	37602	4.88	+2.18401	19%	Uncharacterized protein
S8	I3LMU6	231	37602	4.88	+1.79926	32%	Uncharacterized protein
S9	I3LVD5	377	42108	5.31	+3.5909	34%	Actin, cytoplasmic 1
S10	VIME	408	53692	5.06	+1.76214	36%	Vimentin
S11	F1RP17	246	61756	6.79	+2.05339	19%	T-complex protein 1 subunit gamma
S12	APOA1	177	30307	5.48	-1.72444	40%	Apolipoprotein A-I
S13	I3L954	104	60941	5.55	+1.65533	13%	Uncharacterized protein
S14	A1AT	161	47449	5.54	+21.0393	18%	Alpha-1-antitrypsin
S15	I3LK62	398	28103	6.08	+2.01085	35%	Uncharacterized protein
S16	A6M930	193	46601	5.33	+1.75218	32%	Eukaryotic translation initiation factor 4A isoform 2
S17	B3CL06	446	80 978	7.34	-1.69392	31%	Serotransferrin
S18	A2THZ2	488	71550	5.92	-2.71787	35%	Albumin
S19	TRFE	293	78971	6.93	-5.99885	22%	Serotransferrin
S20	2AAB	104	66913	4.90	-3.77763	19%	Serine/threonine-protein phosphatase 2A 65 kDa regulatory subunit A beta isoform
S21	2AAA	215	66079	5.00	-2.49367	29%	Serine/threonine-protein phosphatase 2A 65 kDa regulatory subunit A alpha isoform
S22	I3L893	501	51044	5.00	-1.78602	41%	Uncharacterized protein
S23	Q9GMA8	156	22883	5.77	-2.36528	19%	Alpha-1-antichymotrypsin 3
S24	L7PBE6	237	60059	5.70	-1.73143	25%	T-complex protein 1 subunit epsilon
S25	I3LD43	152	25581	6.54	-1.67839	27%	Uncharacterized protein
S26	F1RL04	357	47945	4.88	-1.66328	50%	Uncharacterized protein
S27	F1SRK6	216	92633	5.03	-1.64195	14%	Endoplasmin

Abbreviations: MALDITOF/TOF, matrix-assisted laser desorption/ionisation-time-of-flight; Mr, relative molecular mass; PI, isoelectric point; Symbols (+) and (-) denote an increase and a decrease, respectively, in Yorkshire fetal ovaries, when compared with Meishan fetal ovaries.

**Fig 2:**
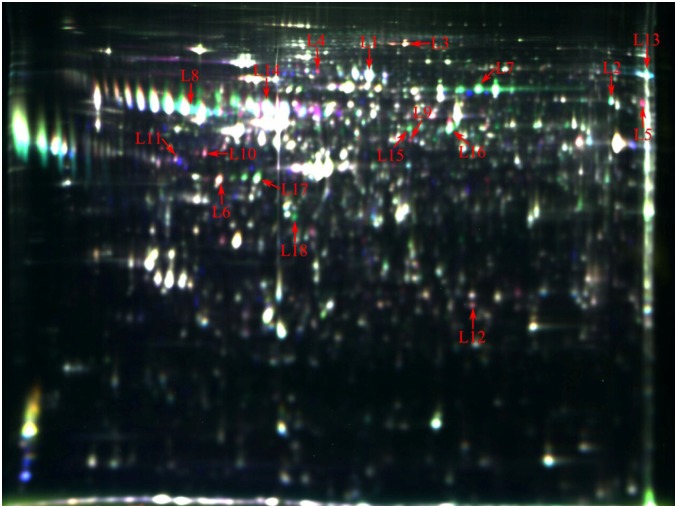
Representative DIGE gel image of differentially expressed proteins in Yorkshire and Meishan fetal ovaries at gestation day 90. The proteins extracted from the Yorkshire and Meishan fetal ovaries on day 90 of gestation samples were labelled with Cy3 and Cy5, respectively. An internal standard protein sample (a mixture of Yorkshire and Meishan fetal ovaries on day 90 of gestation samples) was labeled with the Cy2 dye. The L3-L6 and L9-L12 spots represent up-regulated proteins, while the others spots represent down-regulated proteins in the in Yorkshire fetal ovaries samples compared with the Meishan fetal ovaries samples. The number in the figure corresponds to the number shown in Table 2.

About 1400 protein spots were detected and 42 individual protein spots were significantly changed on day 90 of gestation in Yorkshire and Meishan fetal ovaries. Characterization of the Yorkshire and Meishan fetal ovaries on day 90 of gestation by DIGE analysis is presented in Fig 2 and Table 2. Protein spots with at least 1.6-fold differential expression were selected for MS identification. A total of 16 differentially expressed proteins (7 upregulated, such as heat shock proteins and 9 downregulated, such as alpha-fetoprotein and, serotransferrin) were identified when Yorkshire gilts were compared with Meishan gilts on day 90 of gestation. Other proteins (vimentin, uncharacterized protein where protein-ID is F1SGG2) appeared as multiple spots in DIGE, likely due to their isoforms and/or modifications.

**Table 2:** Differential proteins between Yorkshire and Meishan fetal ovaries on day 90 of gestation identified by MALDITOF/TOF.

No.	Protein-IDs	Moscot score	Mr	PI	Fold change	Coverage	Protein name
L1	FETA	390	70405	5.47	-1.50725	30%	Alpha-fetoprotein
L2	I3LNG8	327	48248	7.01	-2.30613	44%	Uncharacterized protein
L3	I3LQ79	566	99973	5.53	+1.88784	32%	Uncharacterized protein
L4	F1RQU2	120	83543	4.96	+5.03975	18%	Uncharacterized protein
L5	F1RP17	246	61756	6.79	+4.8584	19%	T-complex protein 1 subunit gamma
L6	F1SQ06	150	21243	4.83	+1.91348	31%	Uncharacterized protein
L7	A2THZ2	488	71550	5.92	-3.25783	35%	Albumin (Fragment)
L8	Q9GMA8	156	22883	5.77	-3.04795	19%	Alpha-1-antichymotrypsin 3 (Fragment)
L9	F1S4X9	132	52480	5.64	+3.20847	30%	Uncharacterized protein
L10	VIME	416	53692	5.06	+2.36117	48%	Vimentin
L11	VIME	347	53692	5.06	+1.84908	42%	Vimentin
L12	HSPB1	230	22985	6.23	+1.80479	34%	Heat shock protein beta-1
L13	TRFE	186	78971	6.93	-1.86053	16%	Serotransferrin
L14	A1AT	197	47449	5.54	-1.65057	16%	Alpha-1-antitrypsin
L15	F1SGG2	484	54394	5.70	-1.83149	45%	Uncharacterized protein
L16	F1SGG2	496	54394	5.70	-2.15185	46%	Uncharacterized protein
L17	F1S0J8	460	44186	5.05	-1.65946	51%	Uncharacterized protein
L18	F1RHJ9	205	54675	9.05	-2.41377	22%	Uncharacterized protein

Abbreviations: MALDITOF/TOF, matrix-assisted laser desorption/ionisation-time-of-flight; Mr, relative molecular mass; PI, isoelectric point; Symbols (+) and (-) denote an increase and a decrease, respectively, in Yorkshire fetal ovaries, when compared with Meishan fetal ovaries.

### Functional categories and pathway analysis

**Fig 3:**
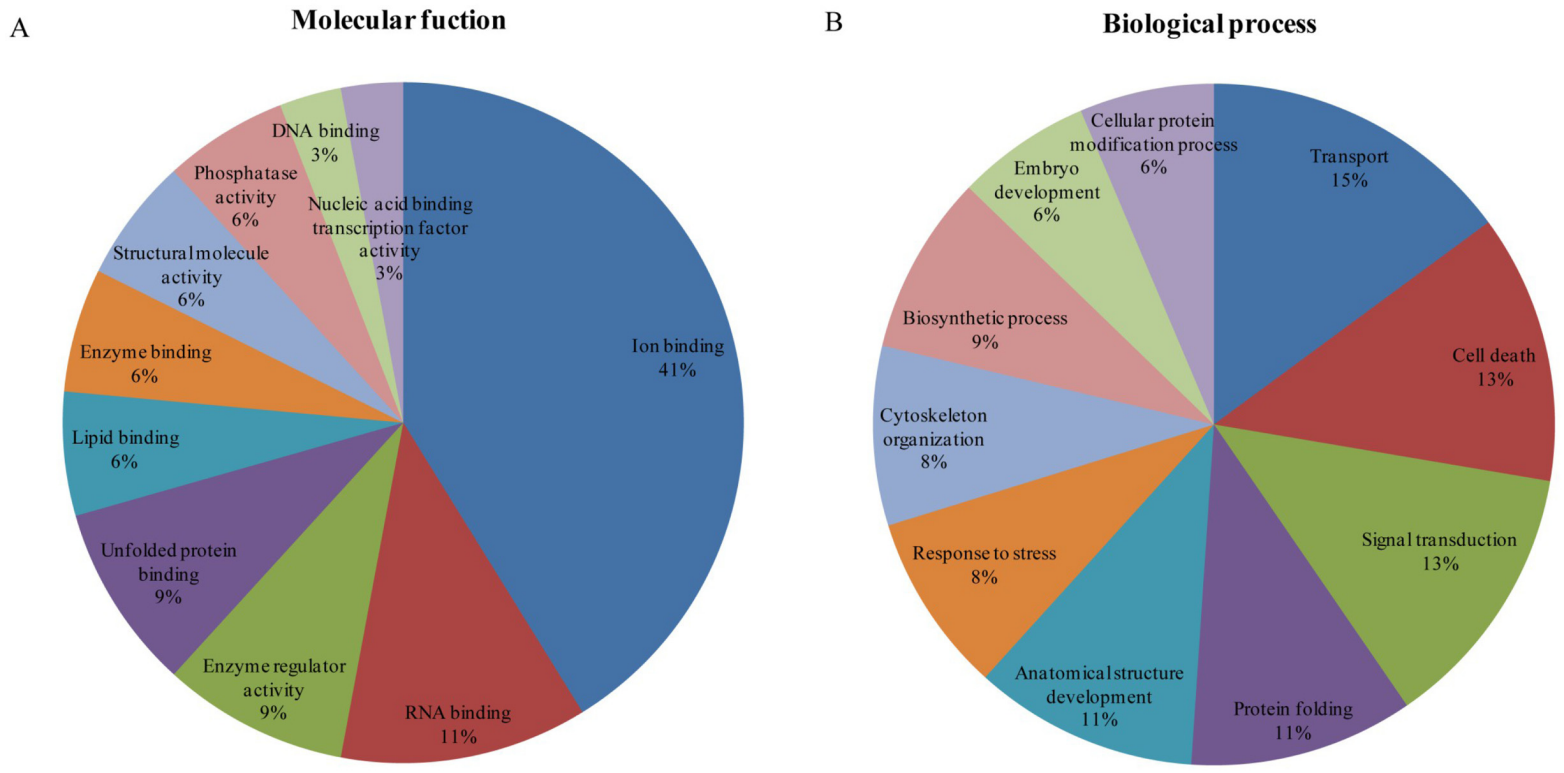
Classification analysis of differentially expressed proteins in Yorkshire and Meishan fetal ovaries at gestation day 55. Ontology analysis of identified Yorkshire and Meishan ovary proteins. The classification of the protein set was performed according to the gene ontology terms: (A) “Molecular function” (19 proteins) and (B) “Biological process” (19 proteins).

To understand the biological and molecular effects of proteins on the development of fetal ovaries in Meishan and Yorkshire gilts, differentially-expressed proteins were characterized by GO annotation. The annotated fetal ovarian proteins were identified using the NCBI and porcine databases and were analyzed by the PANTHER system and GO database. Based on molecular functions, proteins were classified as ion binding proteins (41%), RNA binding proteins (11%), enzyme regulators (9%,), unfolded protein-binding proteins (9%), and lipid-binding proteins (6%), among others (Fig 3A) at day 55 of gestation. The identified proteins were involved in the biological processes of transport (15%) and cell death (13%,), followed by signal transduction (6%), biosynthetic response (9%), and stress response (8%), among others (Fig 3B) on day 55 of gestation. It is interesting that alpha-1-antitrypsin that is associated with enzyme regulators and serine/threonine-protein phosphatase 2A 65 kDa regulatory subunit A beta isoform as well as, serine/threonine-protein phosphatase 2A 65 kDa regulatory subunit A alpha isoform associated with cell death appear to be prominently expressed in Yorkshire gilts on day 55 of gestation.

**Fig 4:**
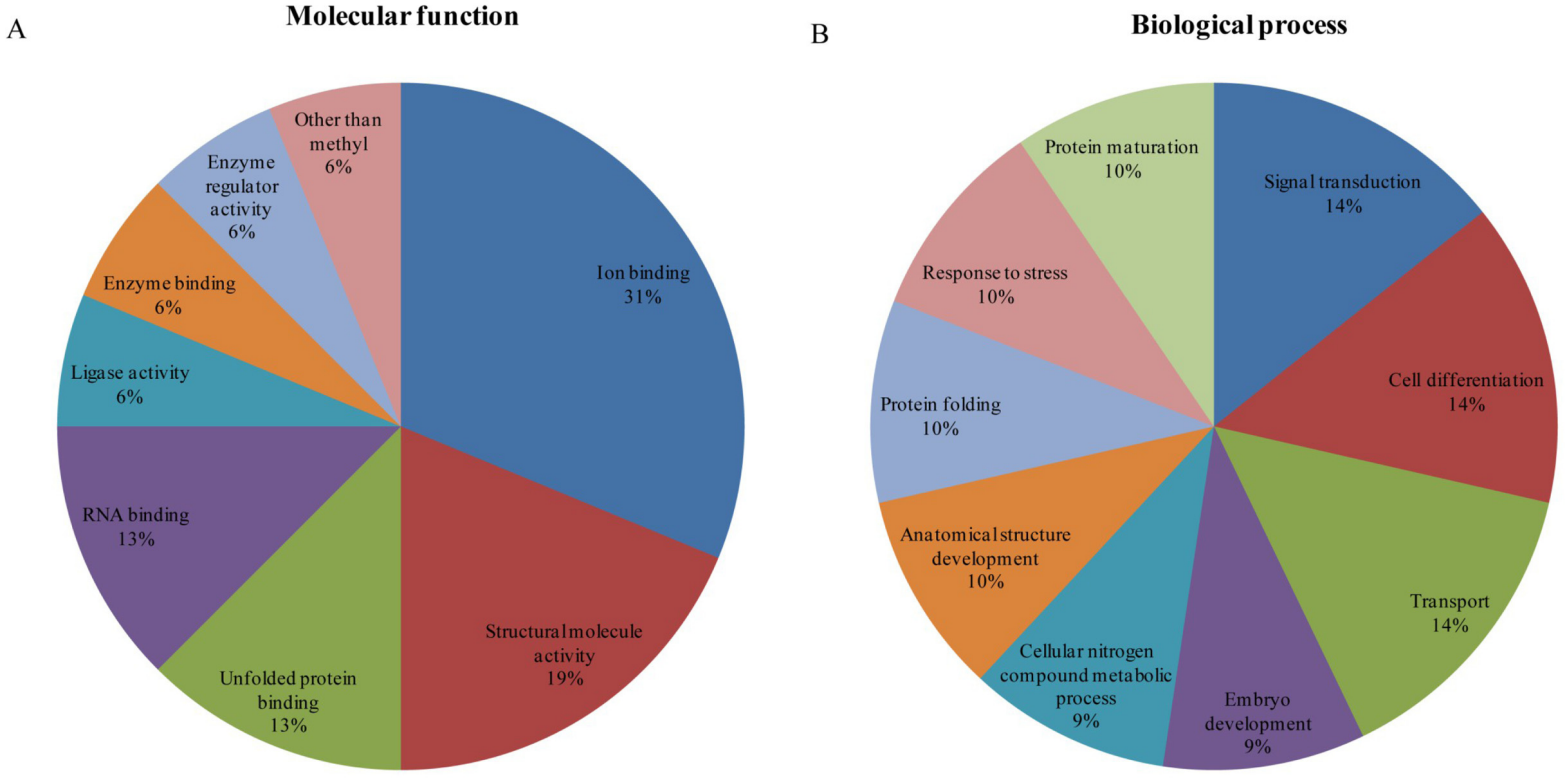
Classification analysis of differentially expressed proteins in Yorkshire and Meishan fetal ovaries at gestation day 90. Ontology analysis of identified Yorkshire and Meishan ovary proteins. The classification of the protein set was performed according to the gene ontology terms: (A) “Molecular function” (11 proteins) and (B) “Biological process” (13 proteins).

Fig 4 demonstrates the diversity of important molecular functions and biological processes of the identified proteins. Fig 4 also displays the corresponding ratio of proteins in Yorkshire and Meishan fetal ovaries at day 90 of gestation. Molecular functions of the identified proteins included ion binding (31%), RNA binding (13%), unfolded protein binding (13%), structural molecular activity (19%) and others (Fig 4A). Similarly, we identified a range of biological processes where the identified proteins were involved, including signal transduction (14%), cell differentiation (14%), transport (14%), embryo development (9%), and stress response (10%), among others (Fig 4B). It is interesting that alpha-fetoprotein and, serotransferrin associated with transport appear to be prominently expressed in Meishan gilts on day 90 of gestation while heat shock proteins associated with stress response appear to be prominently expressed in Yorkshire gilts on day 90 of gestation.

## Discussion

The ovary is one of the most important organs for reproduction [12]. The present study was undertaken with a view to collect information on differentially-regulated phase-specific proteins and their molecular functions that influence fetal ovarian development in Yorkshire pigs and Meishan pigs.

Enrichment analyses of GO annotations were performed for all proteins that were found to be differentially abundant in Yorkshire and Meishan fetal ovaries on days 55 and 90 of gestation (Table 1 and Table 2). These differentially abundant proteins in different breeds, e.g. Hsp90 and Hsp70, may be taken as biological markers for fetal ovary development in future studies. GO analysis revealed differentially abundant proteins regulated various biological processes and molecular functions, including transport, cell death, stress response, cytoskeletal functions, and enzyme regulator activity in Meishan and Yorkshire pigs. Hsp90 and Hsp70 regulation of fetal ovary stress in Meishan pigs is different compared with Yorkshire pigs, indicate a favorable internal environment to stress response in Meishan pig fetal ovaries on day 90 of gestation than in Yorkshire pigs. In particular, we revealed that differentially-expressed proteins were mainly associated with the regulation of primordial follicle assembly and primordial follicle transformation.

GO analysis revealed that most of the differentially abundant proteins were involved in enzyme regulatory activity. Alpha-1-antitrypsin (AAT) is a protease inhibitor that is supposed to finely regulate the balances in protease activities in the ovary. Inactivation of AAT may disrupt the balance of proteolysis/inhibition of proteolysis, and leads to premature follicular rupture and oocyte release [13]. Thus, AAT exerts a key role in follicular maturation and the control of mature oocyte release [14]. In pigs, the majority of AAT is synthesized in the oocyte, but AAT levels were significantly higher in follicles with oocytes compared with uncleaved oocytes [15]. Previous studies found that AAT is a biomarker of oocyte health, which may play an important role in embryo viability and oocyte selection [16]. This is likely to be crucial for obtaining good evidence of proteins synthesized in fetal ovaries. On day 55 of gestation the biological processes that were identified, provided insight into the uncleaved oocyte stage within Meishan pigs and the follicular stage of oocytes in Yorkshire pigs.

The second category of differentially abundant proteins comprise those related to cell death. PP2A is an important and ubiquitously expressed serine threonine phosphatase family, which mediates many fundamental cellular processes in mammalian oocytes, including cell proliferation and apoptosis [17]. The PP2A contains two distinct genes that encode subunits and result in two isoforms called the serine/threonine-protein phosphatase 2A 65 kDa regulatory subunit A alpha isoform and the serine/threonine-protein phosphatase 2A 65 kDa regulatory subunit A beta isoform [18]. PPP2R1A and PPP2R1B regulate ovary activity, G/M transition, and negatively influence mitosis entry [19]. Oocytes entering meiotic or primordial follicle assembly were found to have lower concentrations of PPP2R1A and PPP2R1B. We observed PPP2R1A and PPP2R1B signals in the proteomes of the fetal ovaries, which were stronger in Meishan pigs on day 55, and relatively lower in Yorkshire pigs. The Meishan group is ideal for proteome labeling because PPP2R1A and PPP2R1B concentrations are much higher (3.8-fold and 2.5-fold) than in Yorkshire pigs thus facilitating oocyte nest formation rather than primordial follicle assembly.

We also identified key ovarian proteins that are differentially expressed in Meishan fetal ovaries on day 55 of pregnancy and are involved in physiological processes that occur in the primordial follicle stage of oocyte the assembly. As shown in the oocyte meiosis pathway (S1 Fig) and the PI3K-AKT signaling pathway (S2 Fig), PPP2R1A affected genes in the pathway by delaying reentry into meiosis and has a negative impact on cell survival. The KEGG pathway demonstrated that PPP2R1A is also involved in the TGF-β signaling pathway (S3 Fig), where the dephosphorylation of P70S6K (ribosomal protein S6 kinases) facilitates apoptosis. Thus, we hypothesized that the proteins in Meishan fetal ovaries at day 55 of pregnancy may possibly exist in the germ cells, but not in the follicles, because female pig oocyte nest breaking requires apoptosis that is regulated by relevant proteins. The signal pathways are important regulators of cell survival and apoptosis that may facilitate oocyte nest formation or break. Taking into account the information reported in the paper, the assembly of primordial follicles has not begun in the hypothetical Meishan fetal ovary at day 55 of pregnancy.

GO analysis revealed that the third category of differentially abundant proteins were involved in cytoskeletal organization. The pivotal role of actin in the differentiation of the ovary was observed in all eukaryotes. Actin belongs to a highly conserved family of proteins. In pigs, actin plays a pivotal role in oocyte maturation, fertilization, and embryo development [20]. Abnormal actin distribution in response to cytoskeletal elements of the oocyte results in abnormal cell shape, movement, polarity and embryo quality [20–22]. Therefore, the observed differences in actin abundance, may influence the growth and development of ovaries. Actin is a component of primordial oocytes and plays a crucial role in the migration of spindle fibers [23]. Control over migration of spindle fibers is best known in rat oocytes, in which molecular studies have led to a good understanding of actin involved in spindle fiber migration [24–25]. The main regulator of mouse spindle fiber motility is F-actin, where polymerization around the spindle results in movement [26]. Proteomics data in the current study showed that there was an increased abundance of actin expression (3.6-fold) in Yorkshire fetal ovaries at day 55 of gestation. Taken together, the results indicate that Yorkshire fetal primordial follicles form and enter meiosis at day 55 of gestation.

Initiation of primordial follicle assembly in the ovary due to vimentin is an essential factor that leads to the facilitation of pregranulosa cell motility in ovaries [27]. Meanwhile, vimentin is a typical marker of the ovary [28]. Oocyte nests have a comparatively lower vimentin content compared with those that develop into primordial follicles surrounded by pregranulosa cells [27]. This is particularly remarkable as we achieved specific proteomic data that on day 55 of gestation, where vimentin was substantially altered (Vimentin was 1.76-fold higher in Yorkshire fetal ovary than in Meishan). Previous studies have confirmed that vimentin accelerates the assembly of primordial follicles by facilitating pregranulosa cell motility in ovaries [27]. Meanwhile, the study of fetal ovaries showed that in European pig breeds, primordial follicle assembly was approximately on day 56 of gestation [27]. Similar to previous studies, our results demonstrate that protein labeling of proteomes on day 55 in Yorkshire pigs identified proteins that have a positive effect on primordial follicle formation. Vimentin is thought to promote contact between oocytes and granulosa cells by motile activities and is also an essential indicator of cell survival. Abnormal expression of vimentin results in the reorganization of the cytoskeletal system and finally leads to apoptotic death [29].

The fourth category of differentially abundant proteins comprise those related to stress. Successful ovarian development requires an appropriate internal environment in order to deal with the stress response during the embryo stage. Stress responses play an important role during ovary development in all organisms. Recent reports indicate that heat shock proteins may react to stress conditions [30–31]. We observed that stress-related pathway proteins, such as heat shock protein beta-1, were expressed abundantly in Yorkshire pigs compared with Meishan pigs. The stress-related pathway proteins may play an important role during ovarian growth. By KEGG analysis, we observed that heat shock protein 90 (Hsp90) and heat shock protein 70 (Hsp70) in the ovaries, plays an essential role in oocyte development.

Hsp 90 is considered a predominant member of the Hsp family. Hsp90 isnot only a chaperone essential for the folding and assembly of proteins but plays a key regulatory role in cell cycle, and oocyte maturation [32]. The interactions of Hsp90 between steroid hormone receptors have been investigated in response to stress [33]. Hsp90 is generally increased when cells are exposed to stress. High levels of Hsp 90 can alter ovarian activity. One of the principal functions of Hsp90 is to regulate of reproduction by the reduced expression of estradiol receptors [34]. Considering the above results, it can be concluded that Hsp90 changes can lead to the development of abnormal ovaries.

Hsp70 is found in all eukaryotic cells. An examination of mammalian oocytes, including those of pig, provides important information to understand the constitutive expression of Hsp70 associated with the absence of stress [35–36] and the expression inducible Hsp70 in response to heat stress during oocyte growth [37]. Following thermal stress, Vilma et al observed that the changes in Hsp70 synthesis following heat shock, may depend on the size of oocytes during the developing phase [36]. Moreover, to understand the enhanced resistance of oocytes to heat shock regulated by Hsp70, an experiment of a previous study that use microinjection of Hsp70 mRNA, was performed [38]. In conclusion, Yorkshire fetal ovaries have higher levels of heat shock proteins, suggesting that Yorkshire pigs may require more heat shock proteins for the stabilization of homeostasis. Meishan pigs, on the other hand, are probably not sensitive to the immune effects of heat shock proteins, because the expression of heat shock proteins, is upregulated in response to cellular stress [39]. Taken together, the results indicate a favorable internal environment to stress response in Meishan pig fetal ovaries on day 90 of gestation than in Yorkshire pigs.

Furthermore, extensive studies have revealed an increase in Hsp70 synthesis due to obesity stress that may incite oxidative stress and also plays a critical role in leptin resistance that is connected to the risk of reproductive diseases [40]. It is worth noting that Meishan is considered an ‘obese’ pig breed, but with high fertility [41]. In one research study, the high rate of ovulation and embryo survival, have been described [42]. The current study provides novel findings that the concentration of Hsp70 elevated in the lean breed of Yorkshire compared with obesity Meishan pig. One main difference between these different phenomena is probably that Meishan pigs show an alteration that is also known as leptin resistance, due to the existence of polymorphic leptin receptors [43]. Further evidence supporting the function of Hsp70 is the observation that Hsp70 is positively correlated to leptin [44], indicating that there are multiple mechanisms of Hsp70 regulation in the ovary in Meishan and Yorkshire pigs Therefore, our study provides a new insight into the role of Hsp70 in obese Meishan ovary tissue homeostasis and probably contributes to the low reproductive efficiency reported in obese people.

The fifth category of differentially-regulated abundant proteins identified in this study all play a role in transport. Serotransferrin that are iron-binding transport proteins, are known to have specific functions in granulosa cells, although little is known of their role in follicle development. Serotransferrin may also have a further role in stimulating cell proliferation and suppressing the generation of reactive oxygen species.

Previous experiments were conducted on alpha-fetoprotein (AFP) that is produced by the fetal liver and the yolk sac. AFP is disseminated in the amniotic fluid and passes through the placenta, before being transferred to the ovarian follicles and the oocytes, thus affecting ovarian growth [45]. Catherine et al. demonstrated an interdependency between AFP and estradiol (E2) dynamics in which AFP binds a little E2 produced and inhibits it from initiating the negative feedback at the hypothalamus. This produces relatively high levels of LH and FSH that, bind to LH/FSH receptors on thecal and granulosa cells leading to follicular development [46]. Therefore, this supports the theory that AFP tends to play an essential role in embryonic ovary development. Moreover, to identify normal ovarian growth regulated by moderate concentrations, a previous injection of AFP in normal fetal rats, was performed, observed that normal ovarian cyclic either halted or was nonexistent [45]. In our study, AFP was slightly higher in the Meishan fetal ovary than in the Yorkshire ovary. Following follicle development, AFP levels disappear and, free E2 is available to exert negative feedback [47] that, may cause maternal Meishan pigs to produce relatively high levels of E2 to accelerate follicle growth [48]. Consistent with the data in a previous study, we noticed that AFP levels were lower [49] in postnatal Meishan pigs compared with Yorkshire pigs. Furthermore, Xu et al [50] observed that AFP levels are positively correlated with metabolic syndrome and AFP is an ovarian tumor marker [51], indicating that the regulation of healthy development of ovaries and the high ovulation rate in obese Meishan pigs is through multiple mechanisms of AFP. This is likely to be crucial for obtaining evidence to explicitly demonstrate that the regulation of AFP and E2 in Meishan fetal ovaries on day 90 of gestation provides a good environment for subsequent fetal follicle development, oocyte development, and meaningfully reduces the risk of reproductive disease and increases the ovulation rate of obese Meishan pig.

In this study, we identified several differentially abundant uncharacterized proteins (e.g. protein IDs: F1S0J8, F1SGG2) involved in embryo development, which has an increased abundance of contents at day 90 of gestation ovaries in Meishan pigs. In order to regulate fetal growth, ovaries are equipped with adequate cell differentiation [7]. Currently, little is known about these novel proteins except that they are implicated in embryo development and cell differentiation during ovary development. These uncharacterized proteins may also be ideal candidates for further research in the fetal ovary of Meishan pigs that have greater development potential than Yorkshire pigs. However, further structural and functional analysis of uncharacterized proteins merit investigation.

In conclusion, the proteomes of fetal ovaries are different in diverse biological processes in a time-specific manner in Meishan and Yorkshire breeds. The results also have described several classifications (including transport, cell death, enzyme regulator activity, cytoskeletal framework) of proteins participating in the regulation of fetal ovarian development indicating the major mechanisms responsible for the earlier development of Yorkshire fetal ovaries compared with Meishan fetal ovaries. The Meishan pigs fetal ovaries have a greater developmental potential than the Yorkshire fetal ovaries. Our findings provide a new insight into the decreased expression of Hsp70 on day 90 of gestation and the increased expression of AFP on day 55 of gestation that are likely to play a role in the tissue homeostasis of obese Meishan ovaries and can meaningfully reduce the risk of reproductive disease and increase the ovulation rate.

## Supporting Information

S1 Fig KEGG analysis of PP2A protein regulate oocyte meiosis pathway in Yorkshire and Meishan fetal ovaries at gestation day 55. Fetal ovary metabolic pathway coverage: oocyte meiosis. In the present study, oocyte meiosis signaling pathway highlighting PP2A gene (red) that is differentially expressed in Yorkshire compared to Meishan ovaries at gestation day 55. Adapted from KEGG pathway ssc04114.
(DOC)

S2 Fig KEGG analysis of PP2A protein regulate PI3K-AKT signaling pathway in Yorkshire and Meishan fetal ovaries at gestation day 55. Fetal ovary metabolic pathway coverage: PI3K-AKT signal pathway. In the present study, PI3K-AKT signal pathway highlighting PP2A gene (red) that is differentially expressed in Yorkshire compared to Meishan ovaries at gestation day 55. Adapted from KEGG pathway ssc04151.
(DOC)

S3 Fig KEGG analysis of PP2A protein regulate TGF-β signaling pathway in Yorkshire and Meishan fetal ovaries at gestation day 55. Fetal ovary metabolic pathway coverage: TGF-β signal pathway. In the present study, TGF-β signal pathway highlighting PP2A gene (red) that is differentially expressed in Yorkshire compared to Meishan ovaries at gestation day 55. Adapted from KEGG pathway ssc04350.
(DOC)
