# Supplementary figures and images for: Proteomic Analysis of Fetal Ovary Reveals That Ovarian Developmental Potential Is Greater in Meishan Pigs than in Yorkshire Pigs
Source: PLoS One. 2015 Aug 25;10(8):e0135514. doi: 10.1371/journal.pone.0135514 (PMC4549060; doi:10.1371/journal.pone.0135514)

**
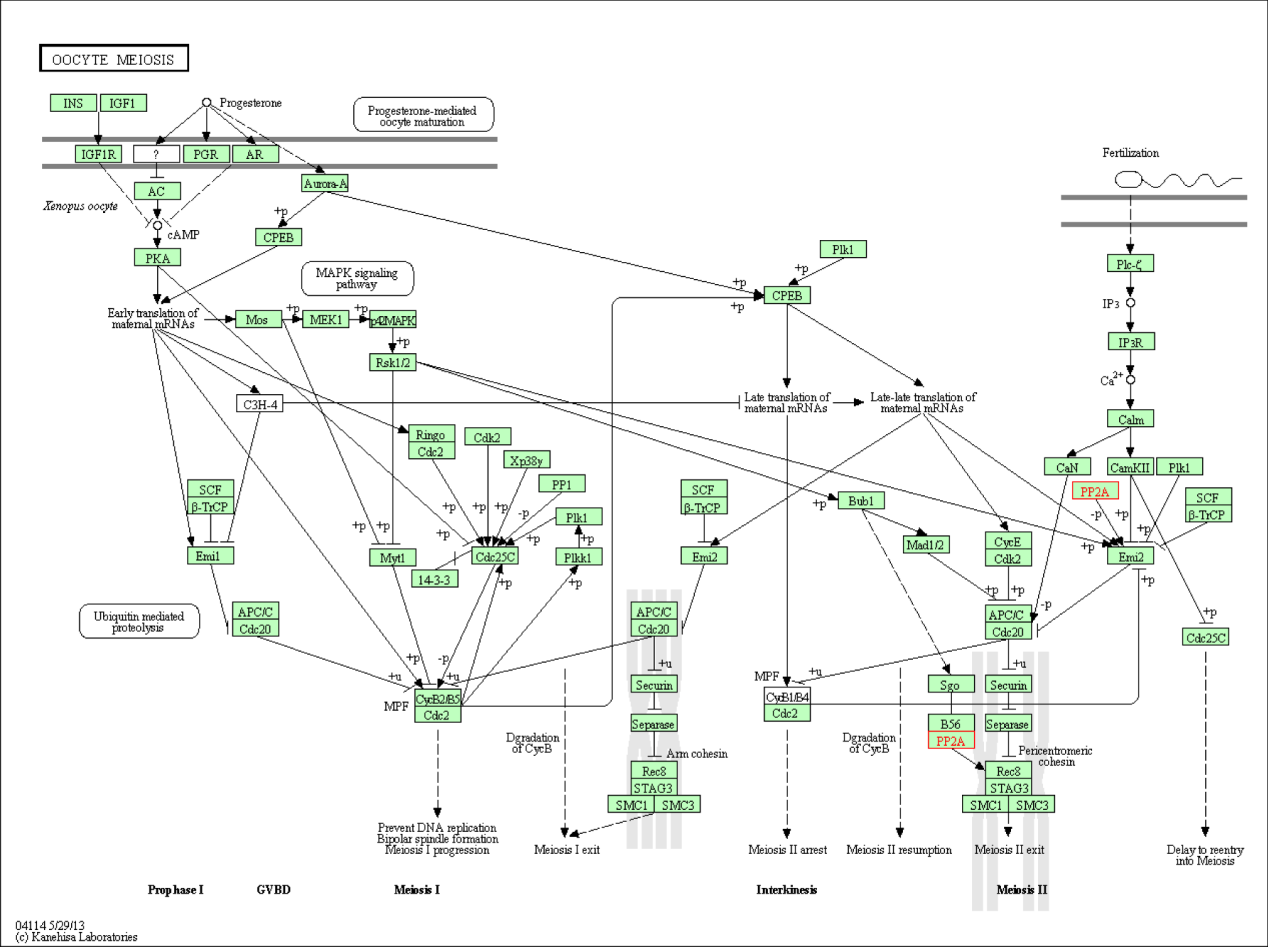
**

**Figure S1**

Supplement: S1 Fig — Fetal ovary metabolic pathway coverage: oocyte meiosis. In the present study, oocyte meiosis signaling pathway highlighting PP2A gene (red) that is differentially expressed in Yorkshire compared to Meishan ovaries at gestation day 55. Adapted from KEGG pathway ssc04114. (DOC) [file pone.0135514.s001.doc]

**
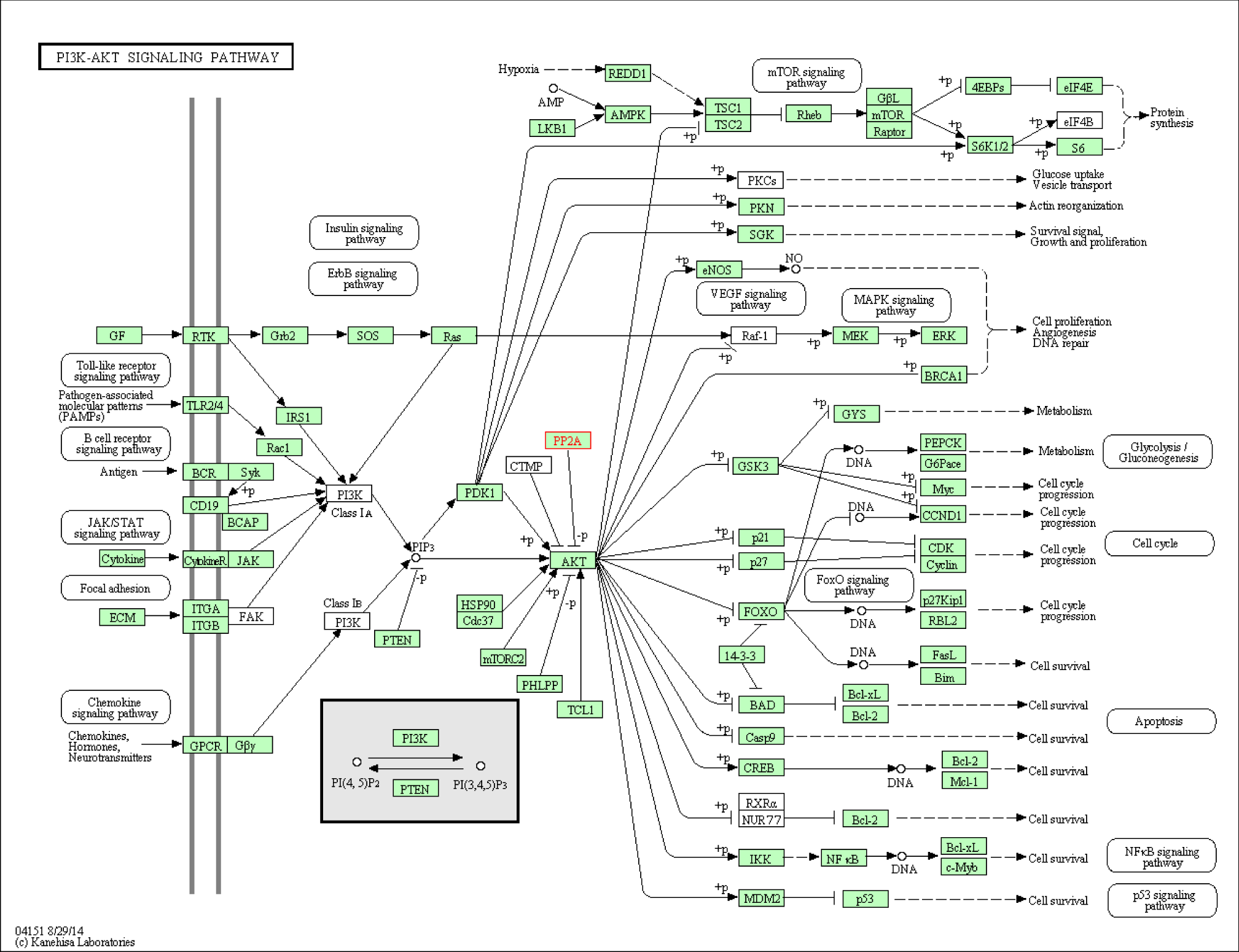
**

**Figure S2**

Supplement: S2 Fig — Fetal ovary metabolic pathway coverage: PI3K-AKT signal pathway. In the present study, PI3K-AKT signal pathway highlighting PP2A gene (red) that is differentially expressed in Yorkshire compared to Meishan ovaries at gestation day 55. Adapted from KEGG pathway ssc04151. (DOC) [file pone.0135514.s002.doc]

**
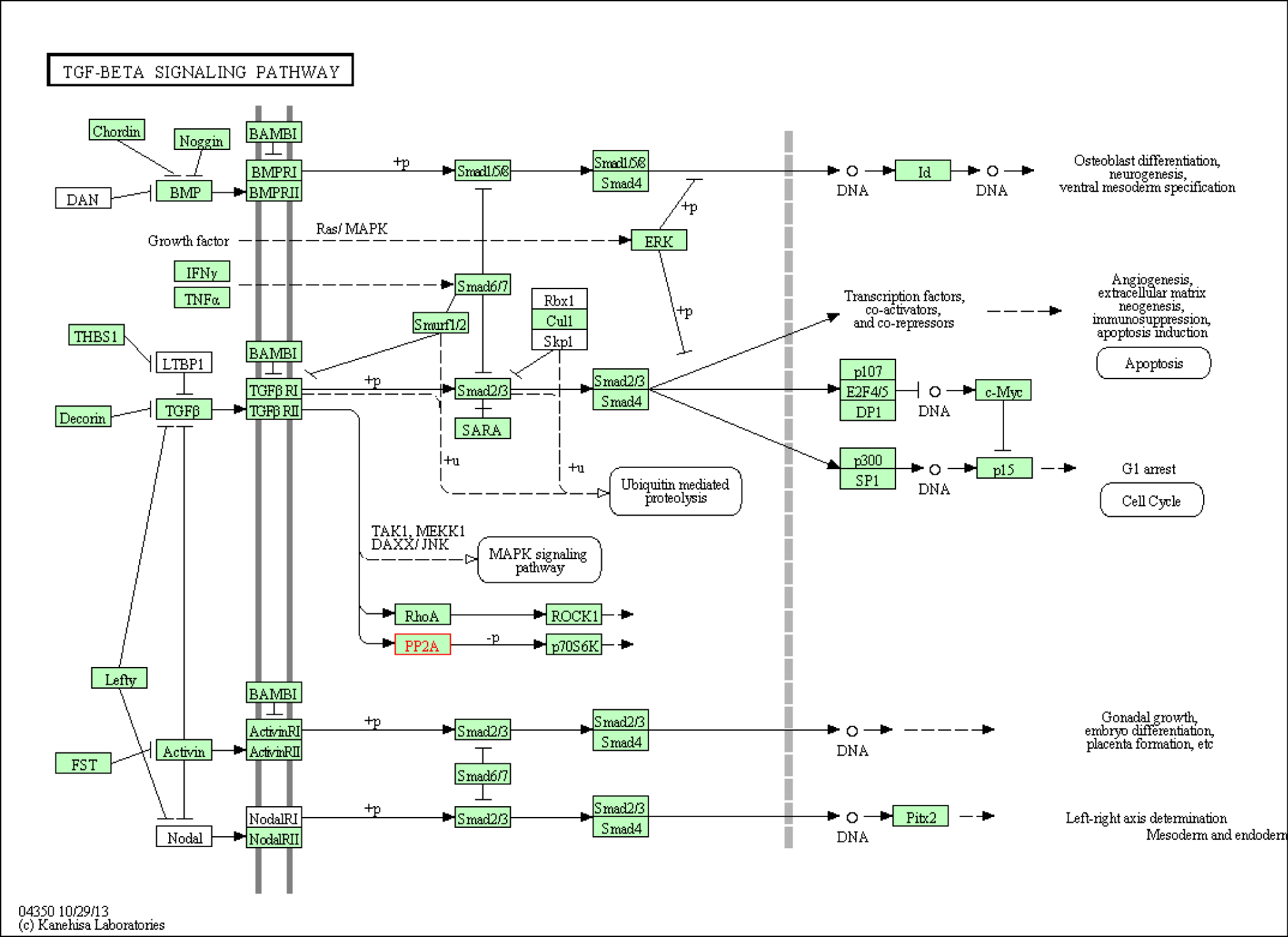
**

**Figure S3**

Supplement: S3 Fig — Fetal ovary metabolic pathway coverage: TGF-β signal pathway. In the present study, TGF-β signal pathway highlighting PP2A gene (red) that is differentially expressed in Yorkshire compared to Meishan ovaries at gestation day 55. Adapted from KEGG pathway ssc04350. (DOC) [file pone.0135514.s003.doc]
